# Orbital angular momentum (OAM) conversion and multicasting using *N*-core supermode fiber

**DOI:** 10.1038/s41598-017-01201-9

**Published:** 2017-04-21

**Authors:** Guang-hao Shao, Shao-cheng Yan, Wei Luo, Guo-Wei Lu, Yan-qing Lu

**Affiliations:** 1 College of Engineering and Applied Sciences, and Collaborative Innovation Center of Advanced Microstructures, Nanjing University, Nanjing, 210093 China; 2 Institute of Innovative Science and Technology, Tokai University, 4-1-1 Kitakaname, Hiratsuka, 259-1292 Kanagawa Japan

## Abstract

We propose and numerically demonstrate a conversion and multicasting scheme of orbital angular momentum (OAM) states by using *N*-core supermode fiber (NCSF), where the topological charges of converted OAM states mainly depend on the injected OAM state and the number of fiber cores. The conversion efficiency (CE) of the converted OAM states could be optimized by properly designing the fiber structure. Take *N* = 6 as an example, ~37% CE could be achieved at telecom bands. Moreover, even for a fabricated NCSF, the CE could be dynamically changed by stretching the fiber or by adjusting the refractive index of the fiber cores through external control of the environmental conditions. Meanwhile, OAM multicasting could also be realized in the designed NCSF. The crosstalk between the multicasted OAM channels and their neighboring ones are assessed to be less than −30 dB. The proposed fiber-based OAM conversion and multicasting system is compatible with the existing optical fiber communication systems, showing potential applications in the future.

## Introduction

In the last decade, optical vortices (OVs) have received considerable research attention. Due to the unique phase and intensity profiles, OVs have been utilized for particle manipulation and microscopy such as stimulated emission depletion (STED)^1–3^. The entanglement of orbital angular momentum (OAM) states of photons has been also demonstrated in quantum information processing^4, 5^. Importantly, the unlimited topological charge values and inherent orthogonality among different OAM states make the OAM state become additional dimension to increase the total system capacity, showing potential applications in optical communication systems. Therefore, it is essential to exploit functionalities for processing OAM states, such as generation, transmission, conversion, multicasting, multiplex/demultiplex and detection. So far, several techniques have been developed for free-space communication systems. In free-space optics, the generation of OAM states has been realized by spiral phase plates, liquid crystal *q*-plates, or fork gratings, while the detection is realized based on beam intensities and profile measurements with commercially available equipment^6–9^. Since OAM normally stays constant in free-space propagation, the transmission of OAM states is relatively simple. A terabit transmission system of multiplexed OAM states has been experimentally demonstrated^10^. The OAM conversion could be realized through nonlinear frequency conversion^11–13^. Moreover, adaptive power-controllable and spatial-mode orbital angular momentum (OAM) multicasting have been experimentally demonstrated^14, 15^. However, in free-space communications based on OAM states, the performance is easily deteriorated by barriers or unstable environments. Therefore, it is still challenging to realize long-distance transmission of OAM states in free-space.

On the other hand, by introducing OAM to optical fiber communication systems, the transmission capacity and spectral efficiency could be further improved. It has been demonstrated that the OAM generation in fiber could be realized using helical fibers, ring fibers, and long-period fiber gratings^16–19^. Through the superposition of high-order modes with a constant phase difference, OAM with a certain phase and intensity distribution could be obtained. For long-distance transmission of OAM states, special micro-structured fibers, such as vertex fibers or multi-ring fibers, have been deployed for its low mode distortion and low crosstalk^20–22^. However, so far only few works have been reported for OAM states conversion and multicasting using fibers^23^, which could indeed provide integrated solution and make the application of OAM states more practical in optical fiber transmission systems.

In this paper, we propose to use an *N*-core supermode fiber (NCSF) for OAM state conversion and multicasting, where the topological charges of the converted OAM states are mainly determined by the injected OAM state and the number of fiber cores. As two of the important metrics for OAM conversion, here, conversion efficiency (CE) of an OAM state is defined as the normalized power weight of the converted OAM state and purity is defined as the normalized power weight left in the originally-injected OAM states (Details see Methods). The simulation results show that with fiber core *N* = 6, ~37% CE is achieved at telecom bands. Since the CE is dependent on the refractive index of fiber cores, it could be dynamically controlled if the fiber cores are filled with a material whose refractive index is sensitive to the external environments. Besides, fiber stretching is another simple and effective way to actively decrease the CEs of the converted OAM states. Meanwhile, the converted OAM spectrum after the NCSF contains several discrete OAM states with the crosstalk less than −30 dB, thus realizing the OAM multicasting functionality. The proposed fiber-based OAM conversion and multicasting system is compatible with and easy to be integrated with optical fiber communication systems, showing potential applications in the future.

## Results

### Design of NCSF

**Figure 1:**
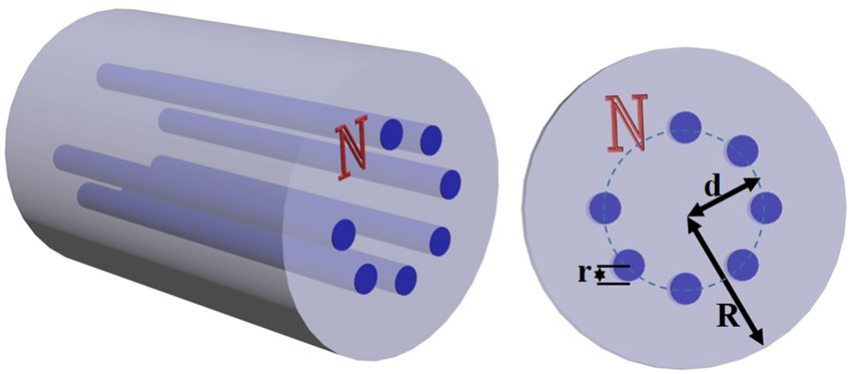
Structure of NCSF. Three dimensional structure and cross-section of an NCSF for OAM conversion and multicasting.

Figure 1 shows the structure of the NCSF proposed for OAM state conversion and multicasting. *N* cores are equally spaced and circularly arranged around the fiber center. *R*, *r*, and *d* represent the radius of the fiber, the radius of the core and the distance between centers of fiber and cores, respectively. It is noteworthy that, compared with the conventional fiber structures, the NCSF has more freedoms in design. In our numerical simulation, the wavelength, *R* and the refractive index of the cladding (*n*_*clad*_) are set to be 1550 nm, 62.5 μm and 1.444, respectively. All of these parameters are consistent with the normal single-mode or multi-mode fiber commonly used in optical fiber communication systems.

### OAM supermodes in NCSF

For an OV showing spiral phase front exp(*ilφ*), each of its photons carries an OAM of *lħ*, where *φ*, *l*, and *ħ* are azimuth angle, topological charge and the reduced Planck constant, respectively. If *l* satisfies 0 < |*l*| < *N*/2, the *l-*th-order supermode is usually quadruple degenerated. Based on the weakly guiding approximation, OAM states could be formed by superimposing two orthogonal supermodes of the same order with a phase difference of π/2^24^. However, the OAM states could not be formed by utilizing other order (|*l*| = 0 or |*l*| ≥ *N*/2) supermodes^25^. Thus, the *l-*th-order (0 < |*l*| < *N*/2) supermode could be defined as the OAM supermode. In other words, the maximum order of the OAM supermodes guided by an NCSF is less than *N*/2.

If an OV carrying OAM_*l*_ (the subscript represents topological charge, 0 < |*l*| < *N*/2) is injected into the NCSF, two orthogonal *l-*th-order OAM supermodes with a phase difference of π/2 could be excited. The injected OV may undergo mode distortions in the fiber and the energy in OAM_*l*_ may distribute to other OAM states. It is found that, although most of the energy stays in OAM_*l*_, the energy also distributes to OAM_*l*±*N*_, OAM_*l*±2*N*_, OAM_*l*±3*N*_, *etc*. (see Methods). Therefore, the converted OAMs with desired states could be expected using the proposed NCSF if an OV carrying OAM_*l*_ is injected.

### OAM conversion in six-core supermode fiber

Without loss of generality, as an example, we set *N* = 6 for further study. First, we assume that the refractive index of the fiber cores (*n*_*core*_) equals to 1.4485. As the maximum |*l*| is less than 3, 2nd-order OAM supermode could be supported and transmitted in the supermode fiber. In another word, if OAM_2_ is injected, the converted 2nd-order OAM supermodes contains states of OAM_2_, OAM_2±1×6_, OAM_2±2×6_, *etc*.

**Figure 2:**
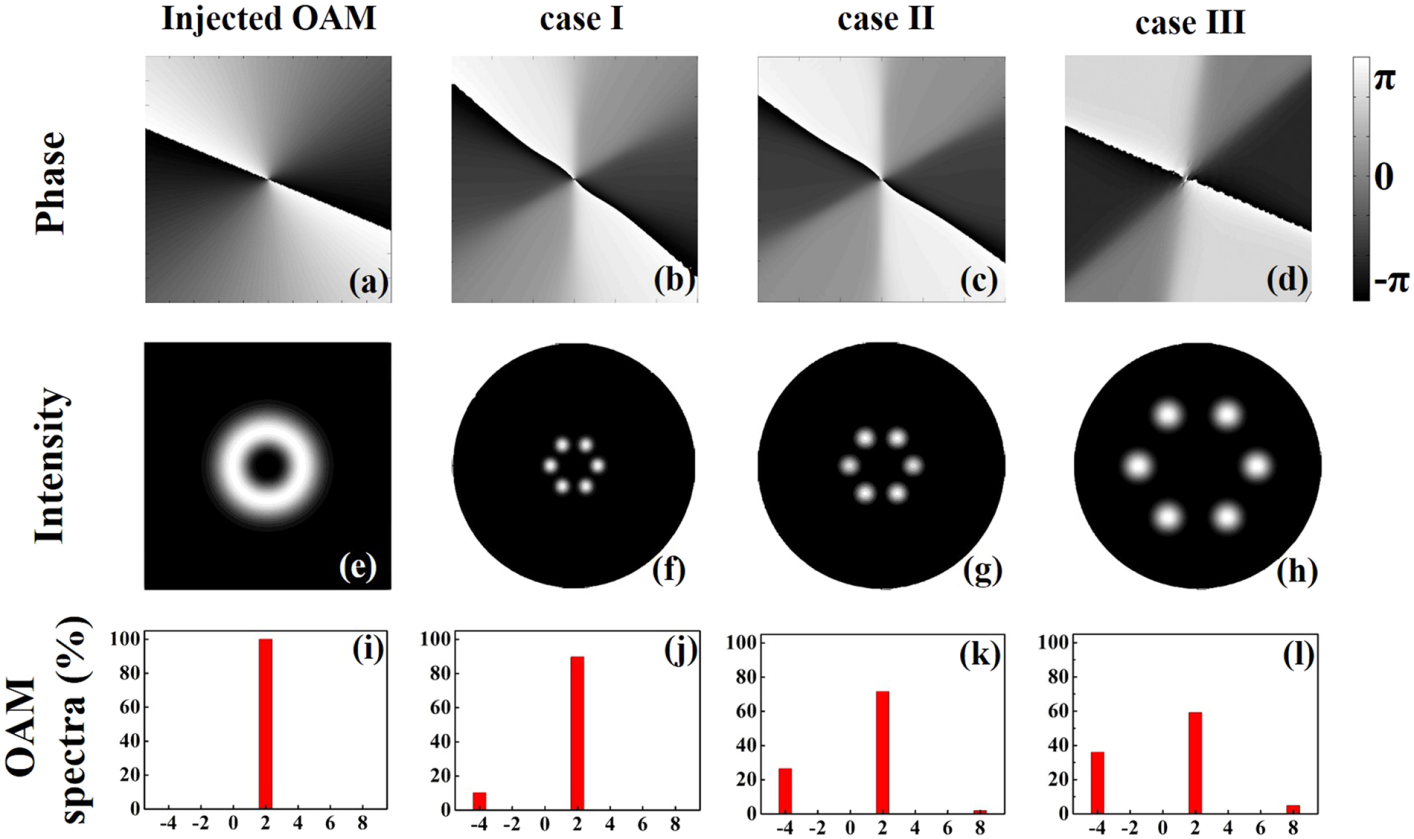
Phase profiles, intensity profiles and OAM spectra. (**a,e,i**) The injected OAM_2_ beam, (**b,f,j**) the transmitted beam for case I (*r* = 4 μm, *d* = 12 μm), (**c,g,k**) case II (*r* = 6 μm, *d* = 16 μm), and (**d,h,l**) case III (*r* = 10 μm, *d* = 30 μm). In Fig. 2(i–l), the x-coordinate and y-coordinate mean topological charge numbers and normalized power weight, respectively.

First, we assume that the refractive index of the fiber cores (*n*_*core*_) equals to 1.4485. Figure 2(a–h) show the phase profiles and intensity profiles of the injected OAM_2_ state and the transmitted OAM states for different values of the parameters *r* and *d*. An abrupt rather than gradual phase shift change in Fig. 2(b–d) indicates the impurity of OAM_2_, *i.e*., the beam may carry more than one OAM states. Here, the converted OAM_−4_ is selected for further analysis. From the OAM spectra shown in Fig. 2(j–l), the CEs of the OAM_−4_ are 10.2%, 26.5%, and 36.0% for case I (*r* = 4 μm, *d* = 12 μm), case II (*r* = 6 μm, *d* = 16 μm), and case III (*r* = 10 μm, *d* = 30 μm), respectively.

**Figure 3:**
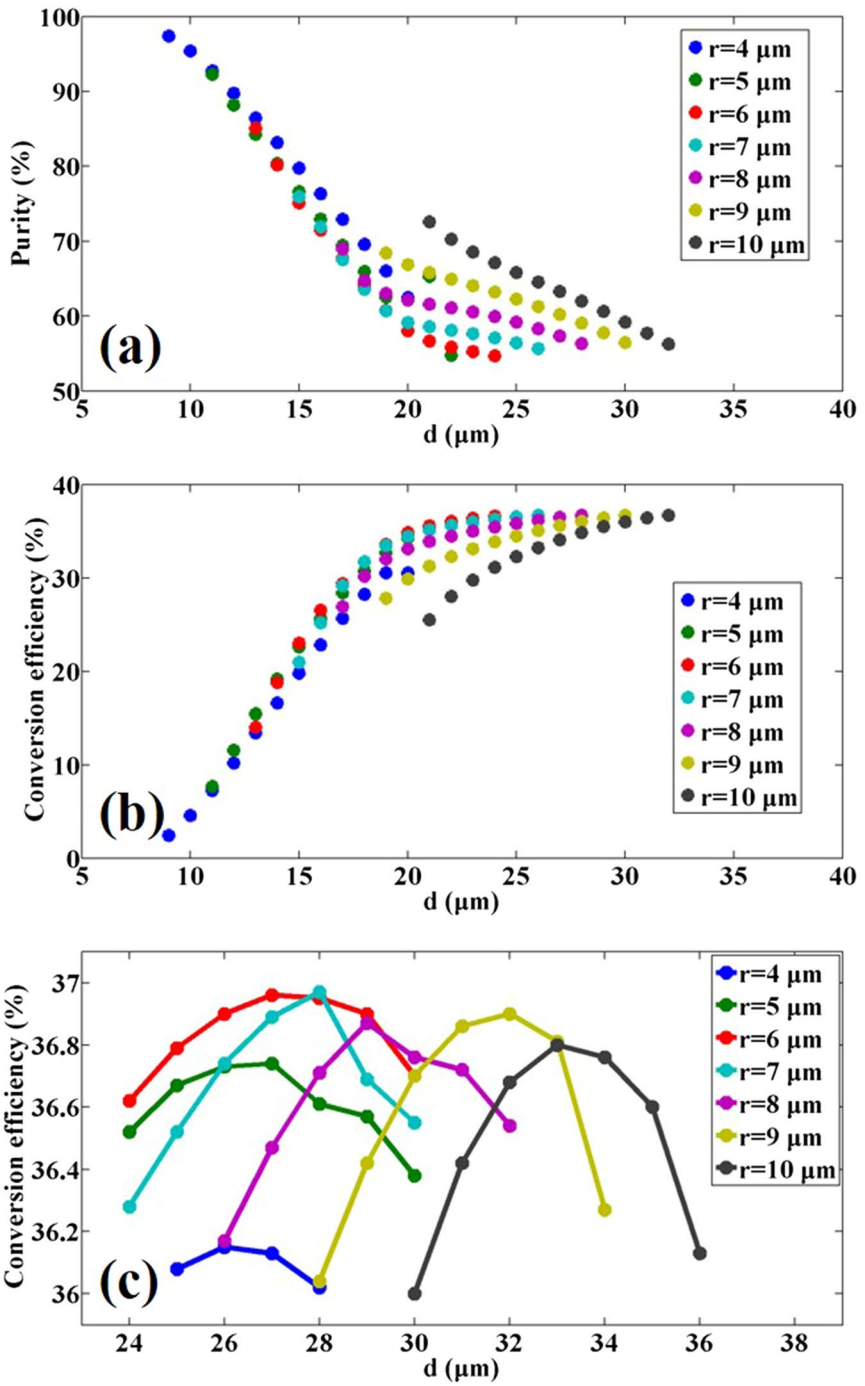
Purity and CE with different structure parameters. (**a**) Purity of the OAM_2_ state and (**b,c**) CE of the OAM_−4_ state in the six-core supermode fiber versus *r* and *d*.

The purity of the injected OAM_2_ and the CE of the OAM_−4_ could be changed by varying *r* and *d*. As shown in Fig. 3, the purity of OAM_2_ varies from ~98.3% to ~54.7% while the efficiency of converted OAM_−4_ varies from ~1.7% to ~37% with different structure parameters. For a larger *d* at a given *r*, the purity of injected OAM state gets lower and the CE of the generated OAM state gets higher simultaneously.

As shown in Fig. 3(b), it seems that the generated OAM_−4_ may have a maximum CE for different parameters of *r* and *d*. If *r* is fixed, the CE increases first and then decreases with the increase of *d*, as shown in Fig. 3(c). There exists a maximum value around 36~37% for each value of *r*. Actually, a stronger coupling between fiber cores would result in a higher purity of the injected OAM state and lower CEs of the converted ones. On the other hand, with a weaker coupling, the CEs become higher while the purity decreases. Therefore, with the increase of *d* at a certain *r*, the cores move away from each other. It makes the coupling between the cores weaker and the purity of the injected OAM state decrease. Meanwhile, the corresponding CE increases and reaches the maximum value. However, when *d* reaches a certain value, the coupling becomes so weak that the fiber could not support OAM supermode anymore. Hence, fiber cores become isolated. In this case, the intensity and phase profile of the transmitted light would be distorted, which makes the supermode fiber not suitable for OAM conversions anymore.

To change the CE, it is not practical to adjust the parameters of the NCSF once it has been fabricated. It will become more practical and attractive if the efficiency of an OAM converter could be dynamically adjusted through active external control. As the cladding is made of SiO_2_ and its refractive index (*n*_*clad*_) is relatively stable in the NCSF, it may be a good approach to adjusting *n*_*core*_ using proper methods for changing the CE.

**Figure 4:**
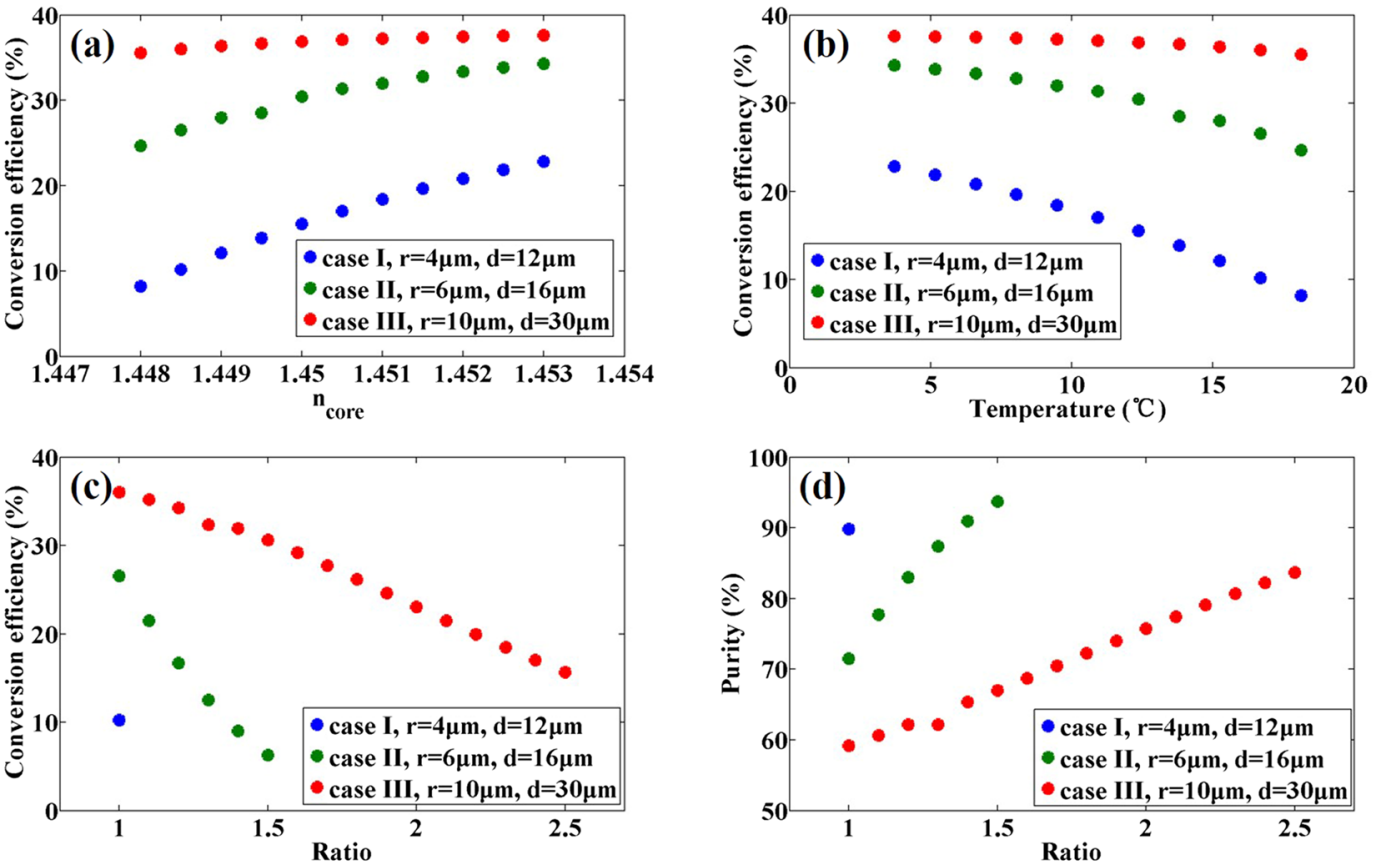
Dynamically controlled purity and CE. (**a**) CE versus *n*_*core*_, (**b**) CE versus temperature if cores are filled with formamide, (**c**) CE of OAM_−4_ state and (**d**) purity of OAM_2_ state versus stretching ratio under the same parameters as in case I (blue), case II (green) and case III (red).

Again, we take the six-core fiber with *R* = 62.5 μm and *n*_*clad*_ = 1.444 as an example. With the OAM_2_ injected to the NCSF, the CE is calculated when *n*_*core*_ changes from 1.448 to 1.453 with parameters (*r* and *d*) set as in the previous cases I, II, and III, as shown in Fig. 4(a).

If the cores are filled with a material whose refractive index is sensitive to the external environments, *n*_*core*_ could be easily changed by tuning the environmental temperature, electric fields, magnetic fields, *etc*. The liquid material could be easily infiltrated into the fiber either through capillary effect or using a syringe. The syringe approach may be more suitable in this case since liquid could be filled over a longer length of NCSF. Moreover, compared to *n*_*core*_ with step values by different doping density, *n*_*core*_ could be continuously changed. For instance, the refractive index of formamide versus temperature approximately satisfies a linear function *n*(*T*) = 1.45429 − 3.47 × 10^−4^ × *T* ^26^, indicating that *n*_*core*_ could be tuned by varying the temperature. When the temperature varies from 3.7 °C to 18.1 °C, *n*_*core*_ changes from 1.453 to 1.448. In addition, *n*_*clad*_ could be seen as constant since the refractive index of SiO_2_ is not sensitive to temperature. With the change of *n*_*core*_, the CE could be changed accordingly, from 8.2% to 22.8%, from 24.7% to 34.3%, and from 35.5% to 37.6%, in cases I, II, and III, respectively. It also shows that the CE is more sensitive to *n*_*core*_ when *r* and *d* are smaller. With larger *r* and *d*, less change in the CE is observed as a function of *n*_*core*_.

Furthermore, even if *R*, *r*, *d*, *n*_*clad*_, and *n*_*core*_ are unchangeable, the CE could also be changed by stretching the fiber using the flame brushing method. The stretching ratio is defined as *R*_ini_/*R*_end_, where *R*_ini_ and *R*_end_ represent the radii of the initial and stretched NCSF, respectively. As shown in Fig. 4, the CE changes significantly apparently as *R*, *r*, and *d* are shrunk in the same ratio. The stretching ratio could not be too large, otherwise the cores may be too small to confine light propagation. In another word, the effective refractive indices of the high-order modes may be lower than *n*_clad_, indicating that the light may leak out from the fiber cladding. For example, with a stretching ratio of 5 for case III, the numerical simulation shows that though the fiber still supports fundamental modes, the effective refractive indices of 1st and 2nd order modes would be 1.44388 and 1.44266, less than 1.444 (*n*_clad_), indicating that the light could not be confined well in the fiber. Since the fiber is operated at 1550 nm, we assume that *r* should better be larger than 4 μm. Under this condition, the largest stretching ratios are chosen as 1, 1.5, and 2.5 for cases I, II, and III, respectively. For cases II and III, the CE of OAM_−4_ state could be changed from 26.5% to 6.3% and from 36.0% to 15.6%, respectively, shown in Fig. 4(c). It also provides a way to improve the purity of the injected OAM state. Shown in Fig. 4(d), the purity of OAM_2_ could be changed from 71.4% to 93.7% and 59.2% to 83.7% for cases II and III, respectively. It is interesting that the purity of OAM_2_ in case II is even lower than that in case I after stretching.

### OAM multicasting using NCSF

In today’s networks, there are some scenarios that require the multi-copy replication of a seed optical signal, which is known as multicasting. In the proposed NCSF, an input OAM_*l*_ mode passes through an *N*-core supermode fiber (*l* should satisfy 0 < |*l*| < *N*/2) and the converted beam may carry several discrete OAM states, *i*.*e*., OAM_*l*±*N*_, OAM_*l*±2*N*_, OAM_*l*±3*N*_, *etc*, realizing the OAM multicasting. Furthermore, since *N* must be larger than 2 to support OAM supermodes, OAM_*l*±*N*_, OAM_*l*±2*N*_, OAM_*l*±3*N*_ are not adjacent. Therefore, the crosstalk between OAM channels and the neighbors is quite low, and measured as less than −30 dB in the numerical simulation.

**Figure 5:**
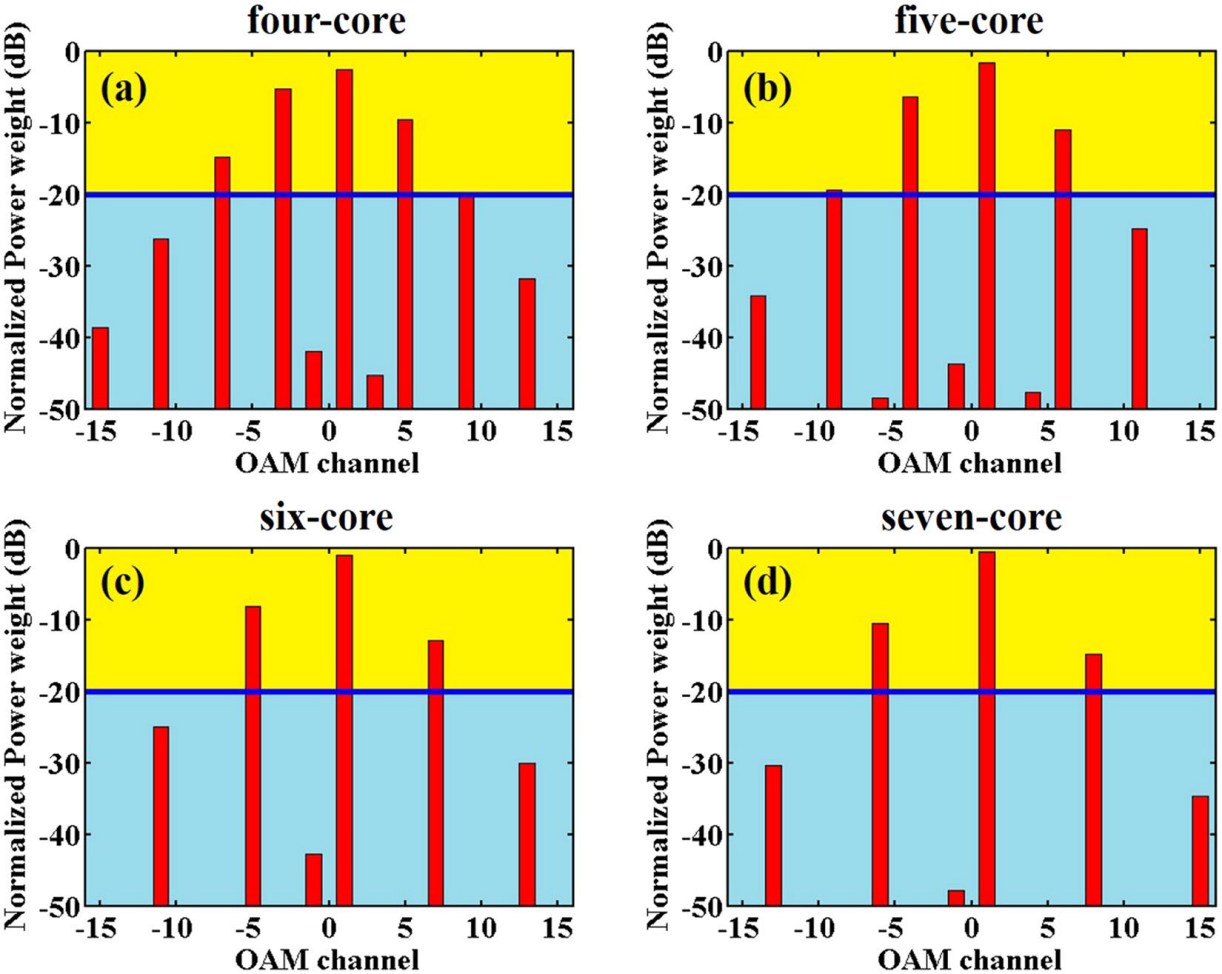
OAM multicasting with different *N*. (**a**) OAM spectrum in four-core supermode fiber. (**b**) OAM spectrum in five-core supermode fiber. (**c**) OAM spectrum in six-core supermode fiber. (**d**) OAM spectrum in seven-core supermode fiber. Normalized power weight under blue line may be not suitable for carrying data.

As mentioned above, different OAM channels could be obtained with different *N*, which could be deployed to realize OAM multicasting with different scales. In addition, since the normalized power weight of each OAM channel could be statically or dynamically changed, the power in different OAM states of output beam could be easily adjusted. Assuming *r* = 5 μm, *d* = 15 μm, *n*_*core*_ = 1.4885 and OAM_1_ is injected into an NCSF, normalized power weights are shown in Fig. 5. If the normalized power weight of an OAM channel is less than −20 dB, it may be too low to carry data. In this case, four channels could be used for OAM multicasting in four- and five-core supermode fiber while three OAM channels could be used in six- and seven-core supermode fiber (yellow background in Fig. 5). The corresponding normalized power of different OAM states are shown in Table 1.

**Table 1:** Normalized power of different OAM channels.

Four-core		Five-core		Six-core		Seven-core	
OAM state	Normalized Power (dBm)	OAM state	Normalized Power (dBm)	OAM state	Normalized Power (dBm)	OAM state	Normalized Power (dBm)
OAM_−7_	−14.8	OAM_−9_	−19.6	OAM_−5_	−8.24	OAM_−6_	−10.6
OAM_−3_	−5.32	OAM_−4_	−6.46	OAM_1_	−1.00	OAM_1_	−0.57
OAM_1_	−2.59	OAM_1_	−1.67	OAM_7_	−12.8	OAM_8_	−14.8
OAM_5_	−9.59	OAM_6_	−11.1	—	—	—	—

## Discussion

In this paper, we have proposed an OAM conversion and multicasting scheme by using an NCSF. The OAM states of the converted beam are determined by the injecting OAM states and the number of cores, and the CE is mainly affected by the radius of the fiber, core size, distance between centers of fiber and cores, and the refractive indices of them. It indicates that the proposed scheme has more freedoms in design than the ring fiber and other conventional fiber structures. For a fabricated OAM converter, the CE and the power weight of OAM channels could be actively changed by tuning *n*_*core*_ with the filled material through the external control or stretching the fiber. Besides, the parameters of the NCSF are similar to the common single-mode or multi-mode fibers, showing compatibility with the existing optical communication system.

Note that, since the OAM states in our proposed conversion system do not undergo frequency conversion, no additional dispersion is introduced during the conversion. With the proper optimization in the supermode fiber design, the topological charge of the converted OAM states could be set far from that of the injected OAM beam and hence larger CE could be realized, which is comparable to the OAM conversion in free optics.

Compared with few-mode fiber (FMF), the mode effective area in our structure is larger, thus leading to a low nonlinear effect. Hence, it could be used in high-power regime such as high power distribution in some scenarios^27, 28^, for example, a power-over-fiber system, where optical power could be delivered through optical fibers. Therefore, our proposed structure may have applications in such scenarios to realize data transmission and power supply simultaneously.

Moreover, if the difference of the effective refractive indices between adjacent order supermodes (Δ*n*_eff_) is larger than 1 × 10^−4^, long distance OAM supermode transmission could be expected with low mode distortion or crosstalk^20, 21^. Thus the OAM supermode, containing the injected and converted OAM states, could transmit in the NCSF, achieving mode division multiplexing. Assume that *R*, *n*_*core*_, *n*_*clad*_ and the wavelength are 62.5 μm, 1.4485, 1.444 and 1550 nm, respectively. Through numerical simulations, as shown in Table 2, some parameters of the NCSF were obtained to satisfy Δ*n*_eff_ ≥ 1 × 10^−4^ for low modal interference. With these parameters, OAM transmission, conversion and multicasting could be realized simultaneously.

**Table 2:** Some parameters supporting long distance transmission of the OAM states.

r (μm)	d (μm)	Δ*n*_eff_ (×10^−4^)			Conversion efficiency of OAM_−4_
		3rd- and 2nd-orders	2nd- and 1st-orders	1st- and 0th-order	
4	9	4.28	5.83	2.67	2.40%
4	10	2.88	4.27	1.96	4.60%
4	11	1.97	3.12	1.44	7.20%
4	12	1.36	2.28	1.07	10.20%
5	11	2.51	3.68	1.59	7.70%
5	12	1.63	2.60	1.15	11.50%
6	13	1.55	2.39	1.02	14.00%

## Methods

### Mode analyses

Any Laguerre-Gaussian (LG) mode with rotational mode number *l* ≠ 0 has a helical wavefront. Therefore, in this work, we use LG modes for free-space OAM analyses. The amplitude of OAM_*l*_ follows

1 $$\begin{array}{c}{u}_{q}^{l}(a,\varphi ,x)\propto {(\frac{a\sqrt{2}}{\omega (x)})}^{l}\exp (-\frac{{a}^{2}}{{\omega }^{2}(x)}){L}_{q}^{l}(\frac{2{a}^{2}}{{\omega }^{2}(x)})\exp (ik\frac{{a}^{2}}{2R(x)})\\ \,\,\,\,\,\,\,\,\,\,\,\,\,\,\,\,\,\,\times \exp (il\varphi )\exp [-i(2q+l+1)\zeta (x)]\end{array}$$

where *l* and *q* are topological charge and radial index of the OAM, respectively. In this work, if OAM_*l*_ injects into the NCSF, two orthogonal *l*-th OAM supermodes are excited. However, such OAM supermodes do not support OV with higher radial index *q*, thus *q* sets to be zero in Eq. (1). ω(x) represents the beam radius at the position of *x*, which is the axial distance from the beam waist. *a* is the radial distance the center axis of the beam and ${L}_{q}^{l}$ is the generalized Laguuerre polynomials. In order to get higher coupling efficiency from free-space to NCSF, the beam waist should be set as the input facet of the NCSF, *i.e*. x = 0. The radial of the beam *ω*(x) thus should approximate to the parameter *d*. *k* = 2π/*λ* means the wave number (in radians per meter). *R*(x) is the radius of curvature of the beam’s wavefronts. *ϕ* represents the azimuthal angle. (2*q* + *l* + 1)·*ζ*(x) is called Gouy phase, which is an extra contribution to the phase^12^.

As for mode analyses in NCSF, we use 2D finite element analysis in the RF Module of COMSOL Multiphysics for numerical simulation. With the assiatnace of the simulator, the effective refractive index and electric fields of OAM supermodes are numerically calculated. The transmitted multi-OAM states are derived from superimposing two orthogonal OAM supermodes of the same order with a phase difference of π/2. Then, the intensity and phase profile of the transmitted beams are obtained. In the fiber core domain, the maximum size of grid is set to be 200 nm to ensure the precision of simulation.

### Normalized power weight of OAM states

The injected OV would undergo mode distortions in the NCSF, and the phase function could be calculated using COMSOL Multiphysics, as described above. We first expand the phase function of an OAM supermode *f (φ)* as

2 $$f(\phi )=\sum _{-\infty }^{\infty }{C}_{p}\exp (ip\phi ),$$

and

3 $${C}_{p}=\frac{1}{2\pi }{\int }_{0}^{2\pi }f(\phi )\exp (-ip\phi )d\phi $$

is the complex coefficient. Therefore, |*C*_*p*_|^2^ is the normalized power weight of the OAM_p_, with $\sum _{p=-\infty }^{\infty }{|{C}_{p}|}^{2}=1$. The integral of *C*_*p*_ in Eq. (3) is approximate to the accumulation of linearly distributed phase from 0 to 2π, that is

4 $${C}_{p}=\frac{1}{M}\sum _{N=1}^{M}f(\frac{2\pi }{M}\cdot N)\exp (-ip\cdot \frac{2\pi }{M}N).$$

*M* sets to be 100 in our calculation and the phase *f*(*φ*) (or $f(\frac{2\pi }{M}\cdot N)$) could be derived from the results of superimposition of two excited orthogonal OAM supermodes.

In this case, |*C*_*l*_|^2^ (if *p* = *l*) is defined as the purity, since it represents the power weight of OAM_*l*_ state in the output beam to that in the input beam. |C_*l*±*N*_|^2^, |C_*l*±2*N*_|^2^, |C_*l*±3*N*_|^2^, *etc*. (if *p* = *l* ± *N*, *l* ± *2* *N*, *l* ± *3* *N*, *etc*.) is defined as the CE of the converted OAM states with topological charges of *l* ± *N*, *l* ± *2* *N*, *l* ± *3* *N*, *etc*.

### OAM supermode

The total field of the superimposed *l*-th OAM supermode can be described as^25^

5 $${X}_{l}\propto \sum _{j=0}^{N-1}\exp [z\,\cos (\phi -{\phi }_{j})+il{\phi }_{j}],$$

where *φ*_*j*_ is the azimuthal angle of *j*-th fiber core and *z* is related to fiber structure. There is a decomposition equation saying

6 $$\exp (z\,\cos \,\alpha )=\sum _{k=-\infty }^{\infty }{J}_{k}(z)\exp (ik\alpha ),$$

where *J*_*k*_(z) is the *k*-th Bessel function. Using Eq. (6), Eq. (5) would be

7 $${X}_{l}\propto \sum _{j=0}^{N-1}\sum _{k=-\infty }^{\infty }{J}_{k}(z)\exp \{i[k\phi +(l-k){\phi }_{j}]\}.$$

Considering the identical relation $\sum _{j=0}^{N-1}\exp (in{\phi }_{j})=N{\delta }_{n,mN}$, where *m* = 0, ±1, ±2, ±3, *etc*.

8 $${X}_{l}\propto N\sum _{m=-\infty }^{\infty }{J}_{mN+l}(z)\exp [i(mN+l)\phi ].$$

In this case, the *l*-th supermode *X*_*l*_ only contains components with helical phase front *exp*[*i*(*mN* + *l*)*φ*]. In another words, it contains OAM_*mN*+*l*_, where *m* = 0, ±1, ±2, ±3, *etc*.
